# Efficacy of adjuvant TACE on the prognosis of patients with HCC after hepatectomy: a multicenter propensity score matching from China

**DOI:** 10.1186/s12885-023-10802-9

**Published:** 2023-04-07

**Authors:** Zhao Wu, Lifeng Cui, Junlin Qian, Laihui Luo, Shuju Tu, Fei Cheng, Lebin Yuan, WenJian Zhang, Wei Lin, Hongtao Tang, Xiaodong Li, Hui Li, Yang Zhang, Jisheng Zhu, Yong Li, Yuanpeng Xiong, Zemin Hu, Peng Peng, Yongzhu He, Liping Liu, Kun He, Wei Shen

**Affiliations:** 1 Department of General Surgery, The Second Clinical Medical College of Nanchang University, The Second Affiliated Hospital of Nanchang University, No.1 Minde Road, Donghu District, Nanchang City, 330006 Jiangxi Province China; 2 Division of Hepatobiliary and Pancreas Surgery, Department of General Surgery, The First Clinical Medical College of Nanchang University, The First Affiliated Hospital of Nanchang University, No. 17 Yongwaizheng Street, Donghu District, Nanchang City, 330006 Jiangxi Province China; 3 Division of Hepatobiliary and Pancreas Surgery, Department of General Surgery, The Second Clinical Medical College, The First Affiliated Hospital, Shenzhen People’s Hospital, Jinan University, Southern University of Science and Technology), No. 1017, Dongmen North Road, Luohu District, Shenzhen City, 518020 Guangdong Province China; 4 Department of Hepatobiliary Surgery, Zhongshan People’s Hospital (Zhongshan Hospital Affiliated to Sun Yat-sen University), No. 2, Sunwen East Road, Shiqi District, Zhongshan City, 528400 Guangdong Province China; 5 School of Public Health, Nanchang University, Nanchang, China; 6 Maoming People’s Hospital, Mao Ming Shiy, China

**Keywords:** Hepatocellular carcinoma(HCC), Hepatectomy, Prognosis, Adjuvant transarterial chemoembolization (TACE)

## Abstract

**Background:**

The survival benefit of adjuvant transarterial chemoembolization (TACE) in patients with hepatectomy for hepatocellular carcinoma (HCC) after hepatectomy remains controversial. We aimed to investigate the survival efficacy of adjuvant TACE after hepatectomy for HCC.

**Methods:**

1491 patients with HCC who underwent hepatectomy between January 2018 and September 2021 at four medical centers in China were retrospectively analyzed, including 782 patients who received adjuvant TACE and 709 patients who did not receive adjuvant TACE. Propensity score matching (PSM) (1:1) was performed to minimize selection bias, which balanced the clinical characteristics of the two groups.

**Results:**

A total of 1254 patients were enrolled after PSM, including 627 patients who received adjuvant TACE and 627 patients who did not receive adjuvant TACE. Patients who received adjuvant TACE had higher disease-free survival (DFS, 1- ,2-, and 3-year: 78%-68%-62% vs. 69%-57%-50%, p < 0.001) and overall survival (OS, 1- ,2-, and 3-year: 96%-88%-80% vs. 90%-77%-66%, p < 0.001) than those who did not receive adjuvant TACE (Median DFS was 39 months). Among the different levels of risk factors affecting prognosis [AFP, Lymphocyte-to-monocyte ratio, Maximum tumor diameter, Number of tumors, Child-Pugh classification, Liver cirrhosis, Vascular invasion (imaging), Microvascular invasion, Satellite nodules, Differentiation, Chinese liver cancer stage II-IIIa], the majority of patients who received adjuvant TACE had higher DFS or OS than those who did not receive adjuvant TACE. More patients who received adjuvant TACE accepted subsequent antitumor therapy such as liver transplantation, re-hepatectomy and local ablation after tumor recurrence, while more patients who did not receive adjuvant TACE accepted subsequent antitumor therapy with TACE after tumor recurrence (All p < 0.05).

**Conclusions:**

Adjuvant TACE may be a potential way to monitor early tumor recurrence and improve postoperative survival in patients with HCC.

**Supplementary Information:**

The online version contains supplementary material available at 10.1186/s12885-023-10802-9.

## Introduction

Hepatocellular carcinoma(HCC) is the most common primary malignant tumor of the liver [1, 2]. Almost half of the newly diagnosed cases of HCC in the world occur in China, resulting in more than 300,000 HCC deaths each year. Hepatitis B virus (HBV) infection is the most important risk factor, and patients with HBV infection slowly progress to the stage of cirrhosis and eventually develop HCC. With the development of medical technology, the current treatment methods for HCC include hepatectomy, radiofrequency ablation, transarterial chemoembolization(TACE), immune targeted therapy, etc. [1–4]. Liver transplantation is often limited by organ shortages, medical technical difficulties and poor medical conditions, which makes hepatectomy remain the first-line treatment for patients with early to mid-stage HCC [2–6]. Because of the majority of HCC in China are diagnosed at an intermediate or advanced stage, the indication of surgery is extended to Chinese liver cancer stage (CNLC) II-IIIa [3, 5–7]. However, the survival outcome of most of these intermediate-advanced patients is not satisfactory [3, 8–10].

There are two patterns of HCC recurrence after curative liver resection: the early-phase and the late-phase [1–3, 7–10]. The early-phase recurrence occurring in liver remnants usually originates from intrahepatic metastasis of the primary tumor, whereas the late-phase recurrence represents de novo lesions in the liver remnant [1–3, 7–11]. As concern the early recurrence, several authors believe that during postoperative adjuvant TACE, residual liver lesions or recurrent tumor lesions can be detected early by angiography, and subsequent administration of local chemotherapeutic agents and embolic agents that block blood supply can kill and inhibit residual or neoplastic tumor cells [12, 13]. However, Chen et al. [13]. found that adjuvant TACE does not delay or prevent tumor recurrence in patients, and its main role is to detect and treat postoperative residual cancer and early recurrent lesions in a timely manner. In addition, the lipiodol used in TACE can be stably deposited in scattered microscopic lesions, which indirectly improves the detection rate of microscopic lesions in postoperative computed tomography (CT) [14]. It is clear that the efficacy of adjuvant TACE for survival of patients with HCC after hepatectomy remains somewhat controversial.

Therefore, the purpose of this study was to investigate the impact of survival with or without adjuvant TACE after hepatectomy in patients with HCC, which hopefully provides a rational treatment decision for clinical work.

## Methods

### Patients

1491 patients with hepatocellular carcinoma who underwent hepatectomy at four medical centers in China from January 2018 to September 2021 were retrospectively evaluated. The above four medical centers are the First Affiliated Hospital of Nanchang University (FAHNU), the Second Affiliated Hospital of Nanchang University (SAHNU), Shenzhen People’s Hospital (SPH) and Zhongshan People’s Hospital (ZPH). The study was conducted in accordance with the Declaration of Helsinki (revised in 2013), approved by the ethics committees of all medical centers, and informed consent was obtained from each patient for the data used in the study. Eligible patients were screened according to the following inclusion criteria: (1) Hepatocellular carcinoma confirmed by postoperative pathology; (2) Tumors were evaluated for CNLC stage I-IIIa; (3) Radical hepatectomy with negative cut margins confirmed by pathology; (4) No antitumor therapy was given before hepatectomy; (5) No past or current history of other malignant tumors; (6) Complete clinical information. Exclusion criteria: (1) Missing clinical data or incomplete follow-up; (2) Preoperative imaging shows lymph node metastasis or extrahepatic metastasis; (3) Postoperative pathology confirmed other non-HCC such as bile duct cancer; (4) Patients who have been diagnosed with other malignant tumors or combined with serious lesions of the brain, heart, lungs and other organs; (5) Patients who died within 90 days after operation. A flow chart of the patients enrolled in this study is shown in Supplementary Fig.S1.

### Hepatectomy and adjuvant TACE

All patients were routinely examined preoperatively with contrast-enhanced ultrasound, enhanced electron CT, and magnetic resonance imaging (MRI), which assessed tumor status and resectable extent. In addition, liver function is assessed with Child-Pugh grading and CT volume measurement in all patients. The methods of liver resection include traditional laparotomy, laparoscopic surgery, anatomic hepatectomy and non-anatomic hepatectomy, and the corresponding surgical methods are adopted according to the location and distribution of the tumor. Among them, anatomic hepatectomy is the complete resection of tumor liver segment or liver segment limited by tumor portal vein branch. Non-anatomic hepatectomy is excision of tumor and partial non-neoplastic liver parenchyma [15, 16].

Patients will be recommended to undergo adjuvant TACE based on high-risk factors affecting patient prognosis, such as concomitant high preoperative tumor marker expression, multiple tumor nodules, tumor diameter > 5 cm, positive MVI, satellite nodules, and poor tumor differentiation [12–14, 17–22]. However, patients will decide whether to follow the advice based on their medical adherence, financial status, or other social factors, which would minimizes patient selection bias. Patients will be routinely examined for liver function, tumor markers, CT and/or MRI to determine tumor recurrence or metastasis before receiving adjuvant TACE. Patients with normal liver function receive adjuvant TACE about 4 weeks after hepatectomy. The Seldinger technique was used to place the hepatic arterial catheter into the proper hepatic artery through the femoral artery and perform TACE on the entire remnant liver. Detection of any suspicious tumor staining in the remnant liver by digital subtraction angiography (DSA) or CT angiography during operation of PA-TACE. If no tumor staining is found, a mixture of chemotherapeutic agents (fluorouracil, epirubicin and platinum) and embolic agents (lipiodol and gelatine sponge) is then administered through a catheter to the remaining liver based on a comprehensive assessment of the patient’s body surface area, physical fitness, and residual liver volume [17–22].

### Follow-up

All patients were followed up during outpatient or inpatient visits. Patients were followed up every 1–2 months for six months after discharge and every 3–6 months thereafter. During follow-up, each patient underwent routine liver function tests, serum alpha-fetoprotein (AFP) analysis, and ultrasound examination. When recurrence is suspected, enhanced CT or enhanced MRI is subsequently used to confirm the diagnosis. Recurrence was defined as neoplastic nodules confirmed by two imaging studies or by needle biopsy. Treatment of recurrent tumors includes liver transplantation, rehepatectomy, local ablation, TACE, chemoradiotherapy, and immunotargeted therapy. Disease free survival (DFS) and overall survival (OS) were used as study endpoints. DFS was defined as the time from hepatectomy to diagnosis of tumor recurrence, while OS was defined as the time from hepatectomy to death or the last follow-up. All patients were followed up to April 1, 2022.

### Propensity score matching

To reduce selection bias and confounding factors, propensity score matching (PSM) analysis was used to eliminate imbalances between groups. A 1:1 nearest neighbor matching algorithm was applied with a caliper width of 0.01. SPSS 26.0 statistical(IBM Corp, Armonk, NY, USA) software was used for PSM.

### Statistical methods

The independent sample T-test was used to detect the continuous data conforming to normal distribution, which was expressed as mean ± standard deviation. Mann-whitney U test was used to detect continuous data with non-normal distribution, which was expressed as median (quartile distance, IQR). Chi-square test was used to detect classified data, which were represented as numbers (n) and proportions (%). Univariate and multivariate analyses were performed in Cox risk models to identify independent prognostic factors for DFS and OS, where variables with P values < 0.05 were used in multivariate analyses in univariate analyses. OS and DFS of independent prognostic factors screened after PSM using kaplan-Meier survival analysis, and differences between curves were estimated by log-rank test. R software (Version 4.2.1 http://www.r-project.org) was used for statistical analysis of the above data. In addition, x-Tiles 3.6.1 software(http://tissuearray.org/) was used to determine the optimal cut-off value for continuous independent prognostic factors screened by Cox proportional risk model after PSM. All P values were obtained by two-tailed test, and P < 0.05 was considered statistically significant.

## Results

### Clinical characteristics

A total of 1491 patients with HCC who underwent hepatectomy were included, including 782 patients who received adjuvant TACE and 709 patients who did not receive TACE. A total of 12 clinical factors [Age, AFP, Alanine aminotransferase (ALT), Aspartate aminotransferase (AST), Alkaline phosphatase (ALP), Platelet-to-lymphocyte ratio(PLR), Operation time, Maximum tumor diameter, Anatomical liver resection, Microvascular invasion (MVI), Differentiation, CNLC stage] were significantly different between groups before PSM (Table 1, all p < 0.05). 627 patients in each group matched by PSM, which resulted in no significant differences in clinical factors between groups (Table 1, all p > 0.05).

**Table 1:** Clinical characteristics of patients with HCC who underwent adjuvant TACE or not

Clinical characteristics		Before PSM				After PSM			
		Total (n = 1491)	Adjuvant TACE		P	Total (n = 1254)	Adjuvant TACE		P
			No (n = 709)	Yes (n = 782)			No (n = 627)	Yes (n = 627)	
**Age (years)**		56.00 (47.00, 64.00)	57.00 (48.00, 66.00)	55.00 (47.00, 63.00)	**0.007**	56.00 (47.00, 64.00)	56.00 (47.00, 65.00)	56.00 (48.00, 64.00)	0.908
**AFP (ng/mL)**		53.05 (6.30, 879.63)	38.40 (5.00, 526.68)	77.200 (7.68, 1000.00)	**0.003**	47.38 (6.00, 718.00)	38.66 (5.01, 620.10)	67.50 (7.05, 812.75)	0.107
**ALT (U/L)**		30.80 (22.00, 45.66)	29.00 (21.00, 43.00)	32.86 (23.00, 48.00)	**0.001**	30.00 (21.20, 44.58)	29.00 (21.05, 44.00)	31.00 (21.45, 44.90)	0.238
**AST (U/L)**		35.00 (26.99, 50.00)	33.21 (26.00, 46.82)	37.00 (28.00, 52.97)	**< 0.001**	34.00 (26.00, 48.00)	33.21 (26.00, 46.73)	34.55 (26.58, 50.00)	0.106
**GGT (U/L)**		53.00 (30.14, 104.31)	53.47 (30.00, 104.90)	52.59 (30.53, 104.00)	0.608	50.06 (29.01, 99.11)	54.63 (30.24, 106.50)	46.54 (29.00, 92.00)	0.083
**ALP (U/L)**		96.00 (75.00, 123.00)	93.00 (72.00, 122.55)	98.13 (77.29, 124.80)	**0.034**	95.00 (75.00, 122.97)	93.23 (73.42, 122.90)	96.50 (76.71, 122.94)	0.327
**TB (mol/L)**		14.60 (10.80, 19.77)	14.10 (10.40, 19.80)	14.80 (11.19, 19.68)	0.106	14.40 (10.70, 19.74)	14.20 (10.40, 19.77)	14.60 (11.07, 19.63)	0.236
**WBC (10** ^**9**^ **/L)**		5.30 (4.27, 6.53)	5.24 (4.22, 6.59)	5.34 (4.33, 6.50)	0.738	5.30 (4.30, 6.50)	5.30 (4.33, 6.65)	5.30 (4.26, 6.44)	0.346
**CR (µmol/L)**		72.80 (62.40, 82.90)	72.70 (62.40, 82.95)	72.86 (62.45, 82.86)	0.829	73.00 (63.00, 83.00)	73.00 (62.45, 82.64)	73.10 (63.13, 83.35)	0.453
**PT (s)**		11.90 (11.30, 12.60)	11.90 (11.30, 12.60)	11.90 (11.30, 12.60)	0.892	11.90 (11.30, 12.60)	11.90 (11.30, 12.60)	11.90 (11.30, 12.70)	0.700
**NLR**		2.20 (1.61, 3.22)	2.20 (1.59, 3.12)	2.22 (1.62, 3.28)	0.194	2.22 (1.61, 3.23)	2.24 (1.60, 3.22)	2.20 (1.62, 3.26)	0.678
**LMR**		3.42 (2.59, 4.77)	3.50 (2.59, 4.90)	3.40 (2.59, 4.72)	0.687	3.45 (2.60, 4.85)	3.47 (2.56, 4.89)	3.44 (2.64, 4.83)	0.495
**PLR**		110.20 (82.18, 153.29)	104.20 (78.01, 148.57)	113.51 (86.96, 158.14)	**< 0.001**	109.78 (82.30, 150.50)	107.20 (80.34, 150.00)	111.71 (85.50, 151.93)	0.153
**Operation time (mins)**		220.00 (165.00, 280.00)	215.00 (160.00, 270.00)	220.00 (176.25, 280.00)	**0.044**	220.00 (170.00, 280.00)	220.00 (165.00, 280.75)	220.00 (175.00, 280.00)	0.680
**Maximum tumor diameter (mm)**		44.00 (27.00, 71.00)	40.00 (25.00, 65.00)	49.00 (29.25, 76.00)	**< 0.001**	43.00 (27.00, 68.00)	43.00 (27.00, 69.00)	43.000 (27.00, 67.50)	0.934
**Gender [n(%)]**	**male**	1263 (84.71)	600 (84.63)	663 (84.78)	0.991	1064 (84.85)	534 (85.17)	530 (84.53)	0.813
	**female**	228 (15.29)	109 (15.37)	119 (15.22)		190 (15.15)	93 (14.83)	97 (15.47)	
**HBV [n(%)]**	**Negative**	199 (13.35)	107 (15.09)	92 (11.76)	0.070	169 (13.48)	94 (14.99)	75 (11.96)	0.137
	**Positive**	1292 (86.65)	602 (84.91)	690 (88.24)		1085 (86.52)	533 (85.01)	552 (88.04)	
**Child–Pugh classification [n(%)]**	**A**	1424 (95.51)	673 (94.92)	751 (96.04)	0.362	1199 (95.61)	596 (95.06)	603 (96.17)	0.408
	**B**	67 (4.49)	36 (5.08)	31 (3.96)		55 (4.39)	31 (4.94)	24 (3.83)	
**Liver cirrhosis [n(%)]**	**No**	373 (25.02)	178 (25.11)	195 (24.94)	0.988	306 (24.40)	155 (24.72)	151 (24.08)	0.844
	**Yes**	1118 (74.98)	531 (74.89)	587 (75.06)		948 (75.60)	472 (75.28)	476 (75.92)	
**Number of tumors [n(%)]**	**single**	1307 (87.66)	630 (88.86)	677 (86.57)	0.207	1104 (88.04)	556 (88.68)	548 (87.40)	0.542
	**multiple**	184 (12.34)	79 (11.14)	105 (13.43)		150 (11.96)	71 (11.32)	79 (12.60)	
**Tumor location [n(%)]**	**left**	472 (31.66)	232 (32.72)	240 (30.69)	0.648	402 (32.06)	207 (33.01)	195 (31.10)	0.768
	**right**	945 (63.38)	444 (62.62)	501 (64.07)		793 (63.24)	391 (62.36)	402 (64.11)	
	**double**	74 (4.96)	33 (4.65)	41 (5.24)		59 (4.70)	29 (4.63)	30 (4.78)	
**Tumor margin [n(%)]**	**Non-smooth**	376 (25.22)	181 (25.53)	195 (24.94)	0.839	313 (24.96)	161 (25.68)	152 (24.24)	0.602
	**Smooth**	1115 (74.78)	528 (74.47)	587 (75.06)		941 (75.04)	466 (74.32)	475 (75.76)	
**Vascular invasion (imaging) [n(%)]**	**Negative**	1362 (91.35)	653 (92.10)	709 (90.66)	0.372	1161 (92.58)	575 (91.71)	586 (93.46)	0.281
	**Positive**	129 (8.65)	56 (7.90)	73 (9.34)		93 (7.42)	52 (8.29)	41 (6.54)	
**Anatomical liver resection [n(%)]**	**No**	457 (30.65)	245 (34.56)	212 (27.11)	**0.002**	384 (30.62)	208 (33.17)	176 (28.07)	0.058
	**Yes**	1034 (69.35)	464 (65.44)	570 (72.89)		870 (69.38)	419 (66.83)	451 (71.93)	
**Laparoscopic surgery [n(%)]**	**No**	898 (60.23)	422 (59.52)	476 (60.87)	0.632	735 (58.61)	365 (58.21)	370 (59.01)	0.819
	**Yes**	593 (39.77)	287 (40.48)	306 (39.13)		519 (41.39)	262 (41.79)	257 (40.99)	
**MVI [n(%)]**	**Negative**	839 (56.27)	450 (63.47)	389 (49.74)	**< 0.001**	741 (59.09)	372 (59.33)	369 (58.85)	0.909
	**Positive**	652 (43.73)	259 (36.53)	393 (50.26)		513 (40.91)	255 (40.67)	258 (41.15)	
**Satellite nodules [n(%)]**	**Negative**	1268 (85.04)	607 (85.61)	661 (84.53)	0.607	1072 (85.49)	527 (84.05)	545 (86.92)	0.173
	**Positive**	223 (14.96)	102 (14.39)	121 (15.47)		182 (14.51)	100 (15.95)	82 (13.08)	
**Differentiation [n(%)]**	**High-medium**	1231 (82.56)	604 (85.19)	627 (80.18)	**0.013**	1038 (82.78)	528 (84.21)	510 (81.34)	0.204
	**low**	260 (17.44)	105 (14.81)	155 (19.82)		216 (17.22)	99 (15.79)	117 (18.66)	
**CNLC stage [n(%)]**	**I**	1259 (84.43)	615 (86.74)	644 (82.35)	**0.026**	1077 (85.89)	540 (86.12)	537 (85.65)	0.162
	**II**	103 (6.91)	37 (5.22)	66 (8.44)		84 (6.70)	35 (5.58)	49 (7.81)	
	**III a**	129 (8.65)	57 (8.04)	72 (9.21)		93 (7.42)	52 (8.29)	41 (6.54)	

**HCC**, Hepatocellular carcinoma; **PSM**, Propensity score matching; **TACE**, Transarterial chemoembolization; **AFP**, Alpha-fetoprotein; **ALT**, Alanine aminotransferase; **AST**, Aspartate aminotransferase; **GGT**, Gamma-glutamyltransferase; **ALP**, Alkaline phosphatase; **TB**, Total bilirubin; **WBC**, White blood cell; **CR**, Creatinine; **PT**, Prothrombin time; **NLR**, Neutrophil-to-lymphocyte ratio; **LMR**, Lymphocyte-to-monocyte ratio; **PLR**, Platelet-to-lymphocyte ratio; **HBV**, Hepatitis B virus; **MVI**, Microvascular invasion; **CNLC**, Chinese liver cancer

### Risk factors for DFS and OS after PSM

**Fig. 1:**
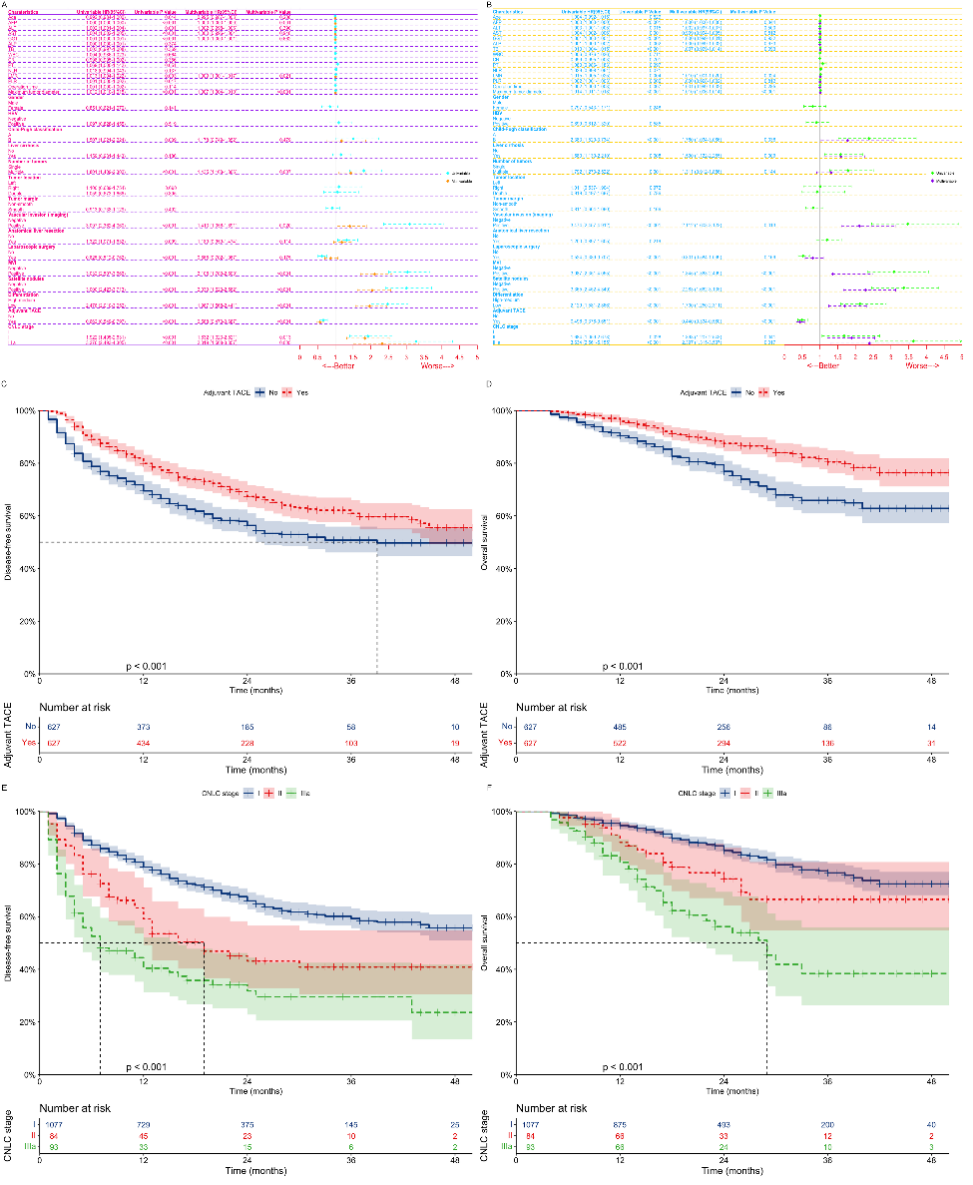
Univariate and multifactorial analysis of Cox regression models for DFS (A) and OS (B) in patients with HCC undergoing hepatectomy after PSM; Kaplan-Meier analysis of DFS (C) and OS (D) for patients with HCC who received adjuvant TACE or not; Kaplan-Meier analysis of DFS (E) and OS (F) for patients with different CNLC stages. **DFS**, Disease-free survival; **OS**, Overall survival; **HCC**, Hepatocellular carcinoma; **PSM**, Propensity score matching; **TACE**, Transarterial chemoembolization; **CNLC**, Chinese liver cancer; **HR**, Hazard ratio; **Cl**, Confidence interval; **AFP**, Alpha-fetoprotein; **ALT**, Alanine aminotransferase; **AST**, Aspartate aminotransferase; **GGT**, Gamma-glutamyltransferase; **ALP**, Alkaline phosphatase; **TB**, Total bilirubin; **WBC**, White blood cell; **CR**, Creatinine; **PT**, Prothrombin time; **NLR**, Neutrophil-to-lymphocyte ratio; **LMR**, Lymphocyte-to-monocyte ratio; **PLR**, Platelet-to-lymphocyte ratio; **HBV**, Hepatitis B virus; **MVI**, Microvascular invasion

10 clinical factors [Fig. 1A; AFP, p = 0.001; Lymphocyte-to-monocyte ratio (LMR), p = 0.021; Maximum tumor diameter, p < 0.001; Number of tumors, p = 0.007; Vascular invasion (imaging), p = 0.020; MVI, p < 0.001; Satellite nodules, p < 0.001; Differentiation, p < 0.001; Adjuvant TACE, p < 0.001; CNLC stage, p = 0.013, p = 0.005] were found to be independent risk factors for DFS, while 10 clinical factors [Fig. 1B; LMR, p = 0.024; Maximum tumor diameter, p < 0.001; Child-Pugh classification, p = 0.038; Liver cirrhosis, p = 0.009; Vascular invasion (imaging), p = 0.013; MVI, p < 0.001; Satellite nodules, p < 0.001; Differentiation, p < 0.001; Adjuvant TACE, p < 0.001; CNLC stage, p = 0.041, p = 0.017] were found to be independent risk factors for OS. During the follow-up period, there were 452 tumor recurrences and 226 deaths after hepatectomy in patients with HCC. Patients who received adjuvant TACE had higher DFS (Fig. 1C- ,2-, and 3-year: 78%-68%-62% vs. 69%-57%-50%, p < 0.001) and OS (Fig. 1D- ,2-, and 3-year: 96%-88%-80% vs. 90%-77%-66%, p < 0.001) than those who did not receive adjuvant TACE (Median DFS was 39 months). There were significant differences in DFS [Fig. 1E; I vs. II, p < 0.001; II (Median of 19 months) vs. IIIa (Median of 7 months), p = 0.012; I vs. IIIa, p < 0.001] and OS [Fig. 1F; I vs. II, p = 0.025; II vs. IIIa (Median of 29 months), p = 0.006; I vs. IIIa, p < 0.001] among patients with different CNLC stages.

### Critical values of continuous variables in risk factors

Among continuous variables, only AFP was an independent risk factor for DFS, so X-Tiles software was used to determine the optimal cut-off value of AFP at 996.7 ng/mL (Supplementary Fig. S2 ABC). Maximum tumor diameter and LMR were the common independent risk factors for DFS and OS among the continuous variables after multi-factor analysis. Therefore, it was determined by x-Tiles software that 2.7 and 2.5 were the cut-off values of LMR (Supplementary Fig. S3 ABCDEF), while 55 and 57 mm were the cut-off values of maximum tumor diameter (Supplementary Fig. S4 ABCDEF). For the convenience of calculation, 2.6 (average) was taken as the optimal cut-off value of LMR, and 55 mm was taken as the best cut-off value of maximum tumor diameter.

### Kaplan-Meier analysis of DFS and OS after PSM

**Fig. 2:**
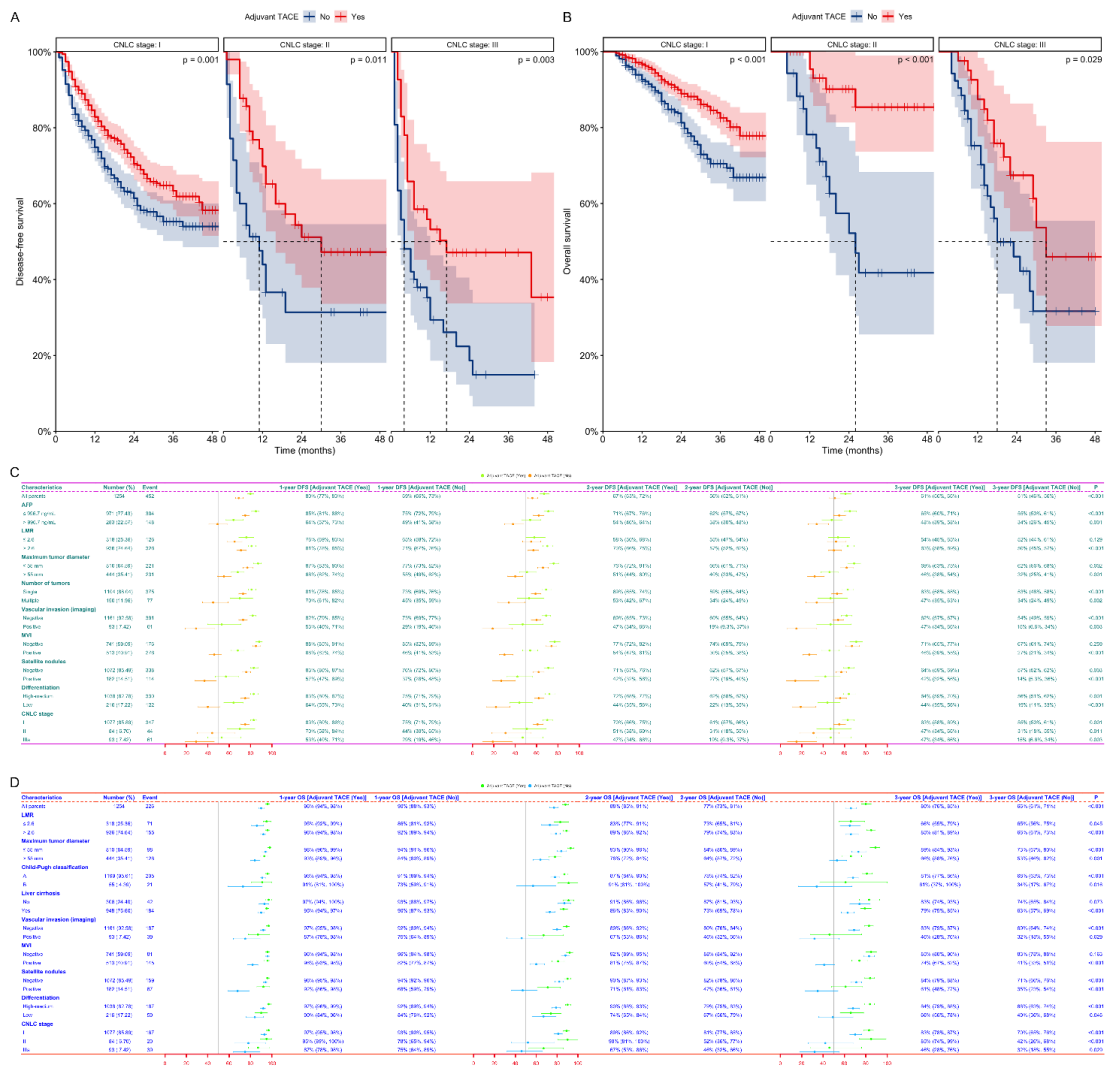
Subgroup Kaplan-Meier analysis of DFS (A) and OS (B) for patients with different CNLC stages who received adjuvant TACE or not; Subgroup forest plots of DFS (C) and OS (D) at 1, 2, and 3 years for patients with different risk factors who received adjuvant TACE or not. **DFS**, Disease-free survival; **OS**, Overall survival; **CNLC**, Chinese liver cancer; **TACE**, Transarterial chemoembolization; **AFP**, Alpha-fetoprotein; **LMR**, Lymphocyte-to-monocyte ratio; **MVI**, Microvascular invasion; **CNLC**, Chinese liver cancer

The results of the subgroup Kaplan-Meier survival analysis at 1, 2, and 3 years were as follows (Fig. 2): Patients at different CNLC stages who received adjuvant TACE had significantly higher DFS [Figs. 2A and I and 83%-70%-63% vs. 75%-61%-55%, p = 0.001; II (Median of 30 months vs. Median of 11 months), 70%-51%-47% vs. 44%-31%-31%, p = 0.011; IIIa (Median of 17 months vs. Median of 4 months), 53%-47%-47% vs. 29%-19%-15%, p = 0.003] and OS [Figs. 2B and I and 97%-89%-83% vs. 93%-81%-70%, p < 0.001; II (No median OS vs. Median of 26 months), 95%-90%-85% vs. 78%-52%-42%, p < 0.001; IIIa (Median of 33 months vs. Median of 18 months), 87%-67%-46% vs. 75%-46%-32%, p = 0.029] than those who did not receive adjuvant TACE. Among the different levels of risk factors affecting prognosis, the majority of patients who received adjuvant TACE had higher DFS [Fig. 2C; AFP (Supplementary Fig. S2 DE; ≤ 996.7ng/mL vs. > 996.7ng/mL, p < 0.001; ≤ 996.7ng/mL, p < 0.001; > 996.7ng/mL, p = 0.001), LMR (Supplementary Fig. S3 GI; ≤ 2.6 vs. > 2.6, p = 0.018; ≤ 2.6, p = 0.129; > 2.6, p < 0.001), Maximum tumor diameter (Supplementary Fig. S4 GI; ≤ 55 mm vs. > 55 mm, p < 0.001; ≤ 55 mm, p = 0.002; > 55 mm, p = 0.001), Number of tumors (Supplementary Fig. S5 AB; Single vs. Multiple, p < 0.001; Single, p < 0.002; Multiple, p = 0.002); Vascular invasion (imaging, Supplementary Fig. S6 AC; Negative vs. Positive, p < 0.001; Negative, p < 0.001; Positive, p = 0.003), MVI (Supplementary Fig. S7 AC; Negative vs. Positive, p < 0.001; Negative, p = 0.259; Positive, p < 0.001), Satellite nodules (Supplementary Fig. S8 AC; Negative vs. Positive, p < 0.001; Negative, p = 0.003; Positive, p < 0.001), Differentiation (Supplementary Fig. S9 AC; High-medium vs. Low, p < 0.001; High-medium, p = 0.001; Low, p < 0.001)] or OS [Fig. 2D; LMR (Supplementary Fig. S3 HJ; ≤ 2.6 vs. > 2.6, p = 0.005; ≤ 2.6, p < 0.001; > 2.6, p = 0.045), Maximum tumor diameter (Supplementary Fig. S4 HJ; ≤ 55 mm vs. > 55 mm, p < 0.001; ≤ 55 mm, p < 0.001; > 55 mm, p = 0.001), Child-Pugh classification (Supplementary Fig. S5 CD; A vs. B, p < 0.001; A, p < 0.001; B, p = 0.016), Liver cirrhosis (Supplementary Fig. S5 EF; No vs. Yes, p = 0.007; No, p = 0.073; Yes, p < 0.001), Vascular invasion (imaging, Supplementary Fig. S6 BD; Negative vs. Positive, p < 0.001; Negative, p < 0.001; Positive, p = 0.029), MVI (Supplementary Fig. S7 BD; Negative vs. Positive, p < 0.001; Negative, p = 0.163; Positive, p < 0.001), Satellite nodules (Supplementary Fig. S8 BD; Negative vs. Positive, p < 0.001; Negative, p < 0.001; Positive, p < 0.001), Differentiation (Supplementary Fig. S9 BD; High-medium vs. Low, p < 0.001; High-medium, p < 0.001; Low, p = 0.046)] than those who did not receive adjuvant TACE.

## Follow-up antitumor therapy

More patients who received adjuvant TACE accepted subsequent antitumor therapy such as liver transplantation (Before PSM, p = 0.037; After PSM, p = 0.017), re-hepatectomy (Before PSM, p = 0.103; After PSM, p = 0.036) and local ablation (Before PSM, p < 0.001; After PSM, p = 0.001) after tumor recurrence, while more patients who did not receive adjuvant TACE accepted subsequent antitumor therapy with TACE (Before PSM, p < 0.001; After PSM, p < 0.001) after tumor recurrence (Table 2).

**Table 2:** Follow-up antitumor therapy after confirmation of tumor recurrence in patients who received adjuvant TACE or not

Anti-tumor therapy	Before PSM			After PSM		
	Adjuvant TACE		P	Adjuvant TACE		P
	No (*n* = 275)	Yes (*n* = 274)		No (*n* = 255)	Yes (*n* = 197)	
**Liver transplantion**	4	12	0.037	4	11	0.017
**Re-hepatectomy**	10	18	0.103	10	17	0.036
**Local ablation**	111	147	< 0.001	105	112	0.001
**TACE**	86	42	< 0.001	79	22	< 0.001
**chemoradiotherapy**	15	8	0.155	14	5	0.125
**immunotargeted therapy**	49	47	0.954	43	30	0.668

**TACE**, Transarterial chemoembolization

## Discussion

Barcelona Clinic Liver Cancer (BCLC) stage has long been a widely used clinical guideline for HCC in international clinical practice [5]. In order to benefit the survival of more patients with intermediate to advanced HCC, the surgical indications of CNLC stage have been expanded compared with BCLC stage [3]. Although the expanded indications for surgery have led to better outcomes for more patients with HCC, these patients inevitably have a higher recurrence rate [3, 8–10]. Therefore, post-hepatectomy adjuvant therapy is more necessary for patients with high-risk recurrent tumors.

In recent years, adjuvant TACE has been the most commonly applied treatment modality for patients with high rates of postoperative tumor recurrence [18–20]. However, adjuvant TACE after hepatectomy has not been fully accepted by experts internationally, and its efficacy is still controversial to some extent. An earlier prospective randomized trial found a significant benefit of liver resection combined with adjuvant TACE in terms of recurrence and survival of patients [21]. In this study, patients who received adjuvant TACE had a significantly improved prognosis, which makes the results consistent with the views of some scholars [18–21]. However, the follow-up period of this study was only 3 years, and the efficacy of adjuvant TACE on the long-term survival of patients still needs to be further explored.

Wang et al. [18] found that the prognosis of patients with MVI who received adjuvant TACE was significantly improved, whereas the prognosis of patients with negative MVI who received adjuvant TACE was not affected. In addition, patients accompanied by satellite nodules and high-medium-differentiated tumors also obtained better survival effects after receiving adjuvant TACE. However, patients with Low differentiated tumors in this study who received adjuvant TACE did not have a longer survival. This result above may be related to the overpowering invasive and metastatic ability of Low differentiated tumors [22]. Thus, it seems that the postoperative pathological findings are highly informative for patients to choose adjuvant TACE or not.

Whether the indication for postoperative adjuvant TACE depends exclusively on the high-risk recurrence population. It is well known that vascular tumor invasion is a high-risk factor that severely affects patient prognosis [23, 24]. In this study, patients with or without vascular tumor invasion had better survival results after receiving adjuvant TACE. In addition, patients in different CNLC stages also have a survival benefit with adjuvant TACE. It follows that the population suitable for adjuvant TACE is not limited to such patients. Meanwhile, some scholars believe that tumor recurrence in patients with cirrhosis may be a new tumor rather than an intrahepatic metastasis of the tumor, which may be the result of surgery to remove only the primary tumor but not the sclerotic liver with cancerous potential [25, 26]. Marasco et al. [26] showed that cirrhosis is a risk factor for late recurrence of HCC and is directly related to the degree of liver disease and portal hypertension. Patients with cirrhosis in this study had a significantly lower OS rate than those without cirrhosis. Interestingly, OS improved in patients with and without cirrhosis who received adjuvant TACE. It is worth considering whether patients without high-risk factors should receive adjuvant TACE.

It should not be overlooked that adjuvant TACE is not only a treatment but also an invasive diagnostic procedure. If tumor staining is detected during adjuvant TACE, it may be considered a diagnostic tool to monitor tumor recurrence rather than an adjuvant treatment [27]. On the other hand, more patients who received adjuvant TACE underwent subsequent curative treatment (liver transplantation, re-hepatectomy, or local ablation) after the diagnosis of tumor recurrence, which may have led to longer overall survival for them. Earlier monitoring of tumor recurrence during adjuvant TACE may result in lesions that are usually localized and controllable, which further affects subsequent antitumor therapy. In contrast, more patients who did not receive adjuvant TACE underwent subsequent palliative treatment (TACE), which may be related to the greater extent of tumor recurrence and unfavorable factors such as large vessel cancer thrombosis and extrahepatic metastases [28]. In conclusion, the effect of adjuvant TACE on recurrence patterns and the direct therapeutic effect of adjuvant TACE itself may together contribute to the survival benefit in this patient population. This may explain why the majority of patients in this study had a significant survival benefit after receiving adjuvant TACE.

Several limitations of this study should be noted: (1)As a retrospective analysis, patient selection bias could not be completely avoided; (2)There are still no formal clinical guidelines for adjuvant TACE, and the drugs and doses may vary from one medical center to another; (3) Ultrasound combined with AFP was used as the initial monitoring tool for follow-up in this study, which had an impact on the rate of early recurrence of missed tumors. It is hoped that more large, multi-center, prospective trials will be conducted in the future to verify the findings of this study.

Overall, adjuvant TACE is not only an invasive diagnostic procedure for early monitoring of tumor recurrence, but may also be a potential treatment to improve the survival of patients with HCC after hepatectomy.

## Electronic supplementary material

Below is the link to the electronic supplementary material.

Supplementary Material 1

Supplementary Material 2

Supplementary Material 3

Supplementary Material 4

Supplementary Material 5

Supplementary Material 6

Supplementary Material 7

Supplementary Material 8

Supplementary Material 9

Supplementary Material 10

## Data Availability

The datasets generated and analyzed during the current study are not publicly available due to privacy and ethical concerns, but are available from the corresponding author on reasonable request.
