# Supplementary figures and images for: Efficacy of adjuvant TACE on the prognosis of patients with HCC after hepatectomy: a multicenter propensity score matching from China
Source: BMC Cancer. 2023 Apr 7;23:325. doi: 10.1186/s12885-023-10802-9 (PMC10080834; doi:10.1186/s12885-023-10802-9)

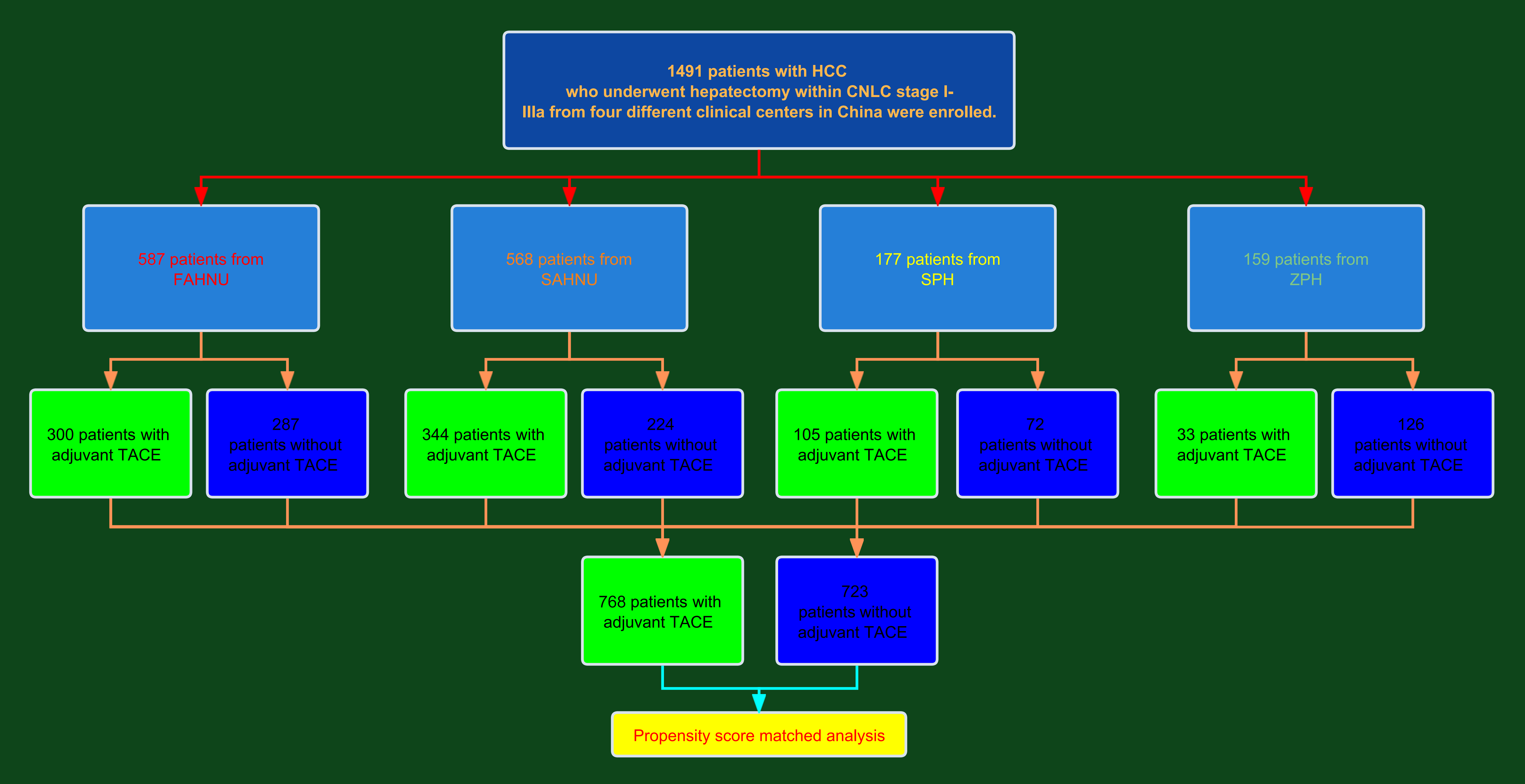

Supplement: Supplementary file 1 — Supplementary Material 1 [file 12885_2023_10802_MOESM1_ESM.tif]

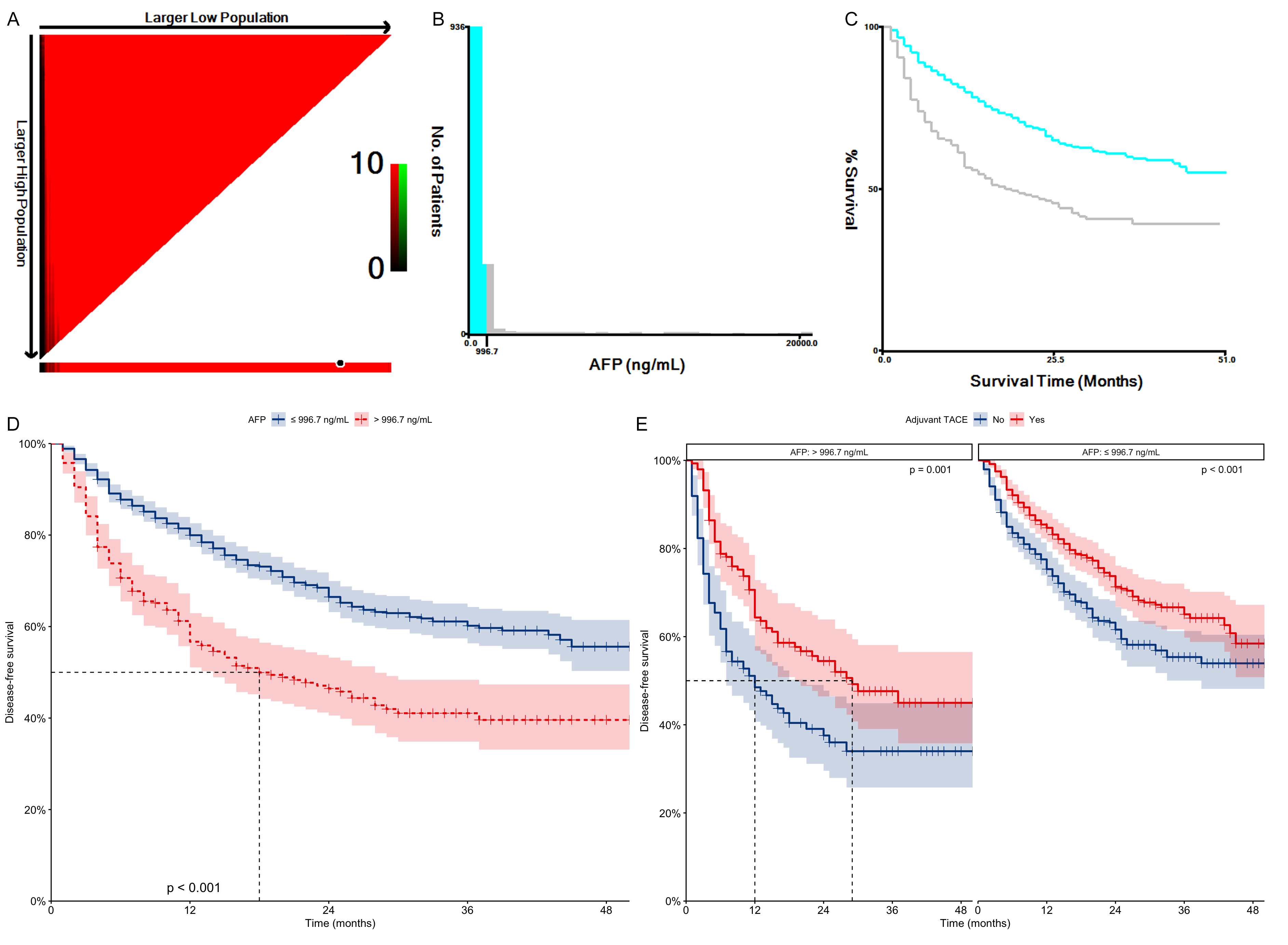

Supplement: Supplementary file 2 — Supplementary Material 2 [file 12885_2023_10802_MOESM2_ESM.tif]

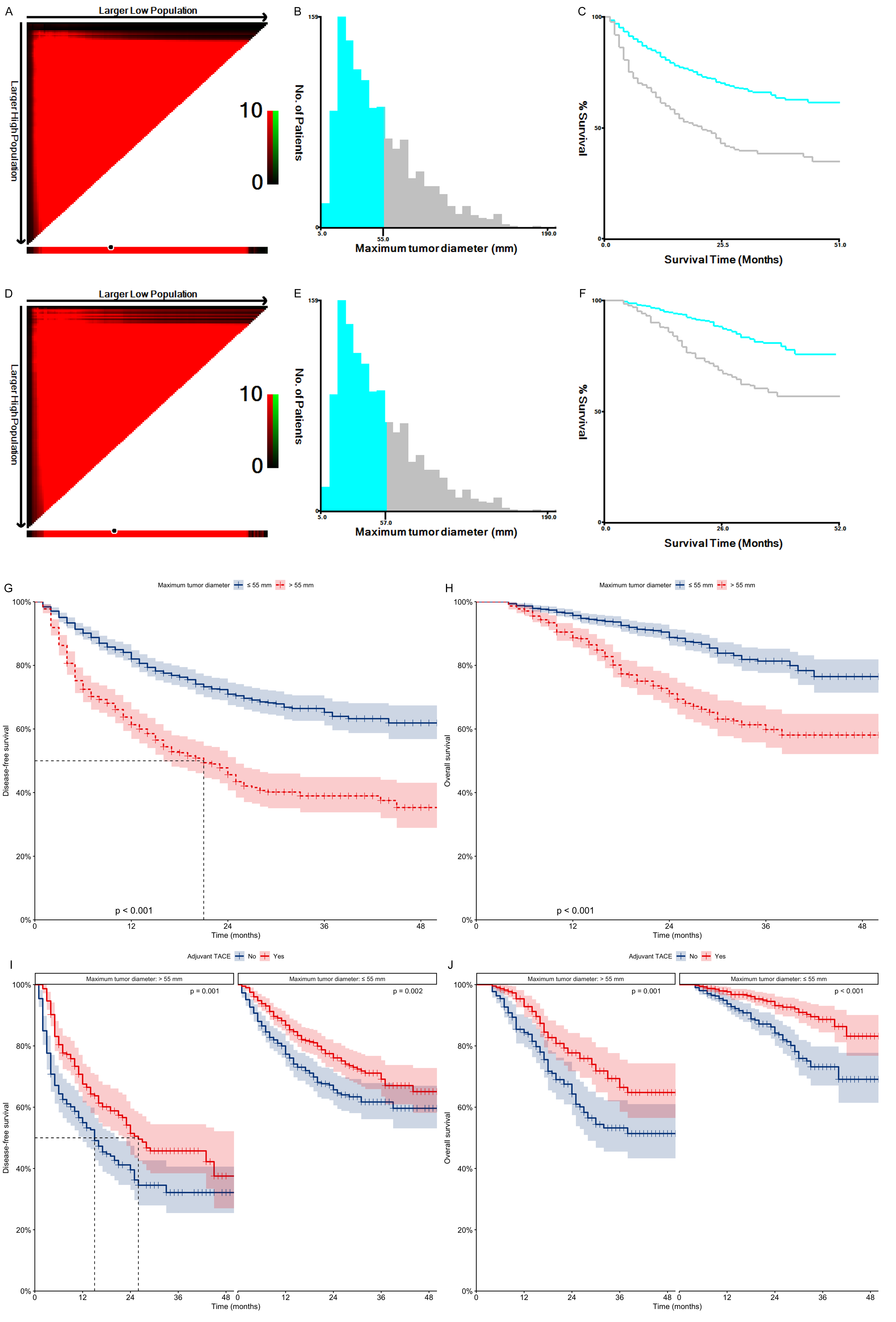

Supplement: Supplementary file 4 — Supplementary Material 4 [file 12885_2023_10802_MOESM4_ESM.tif]

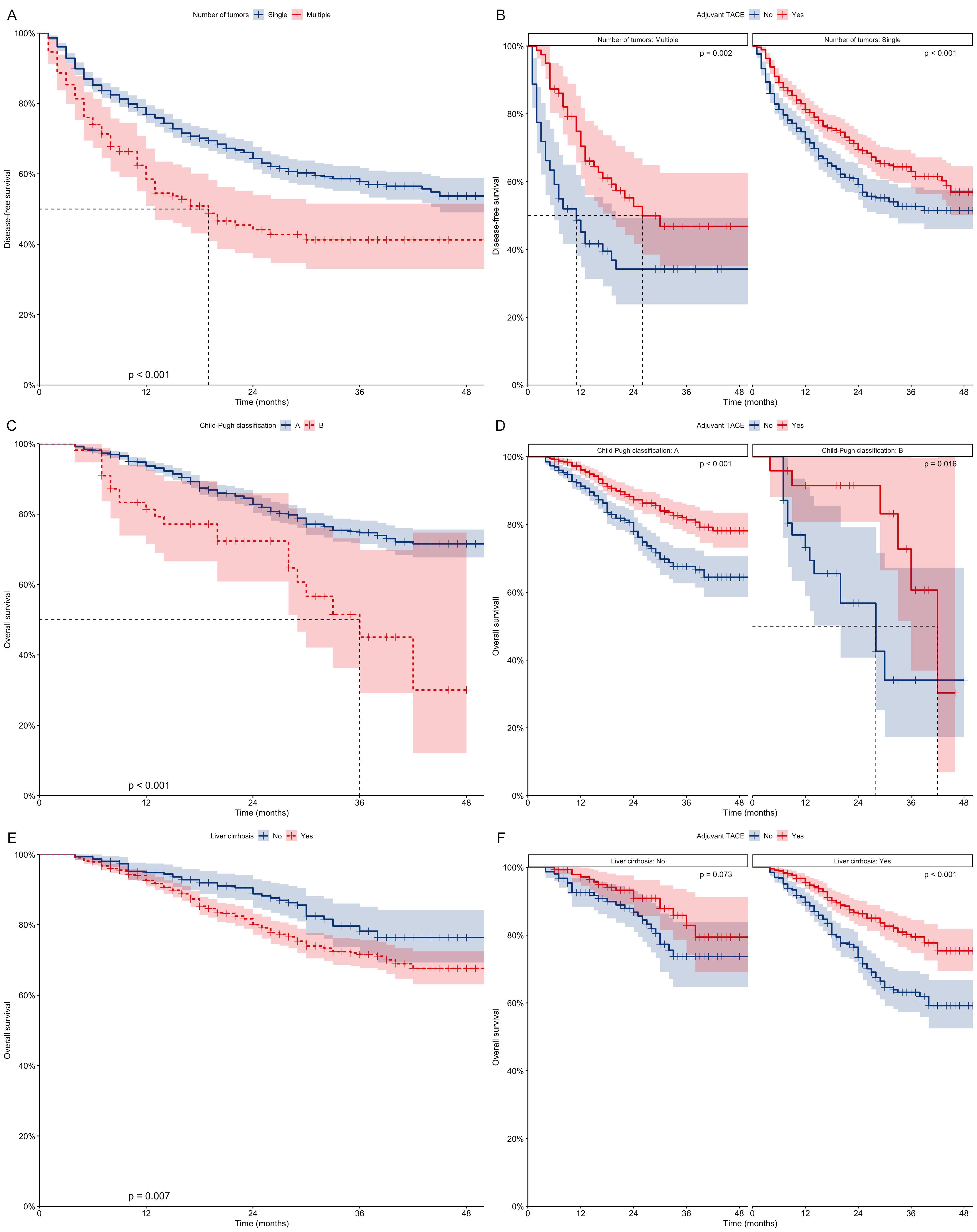

Supplement: Supplementary file 5 — Supplementary Material 5 [file 12885_2023_10802_MOESM5_ESM.tif]

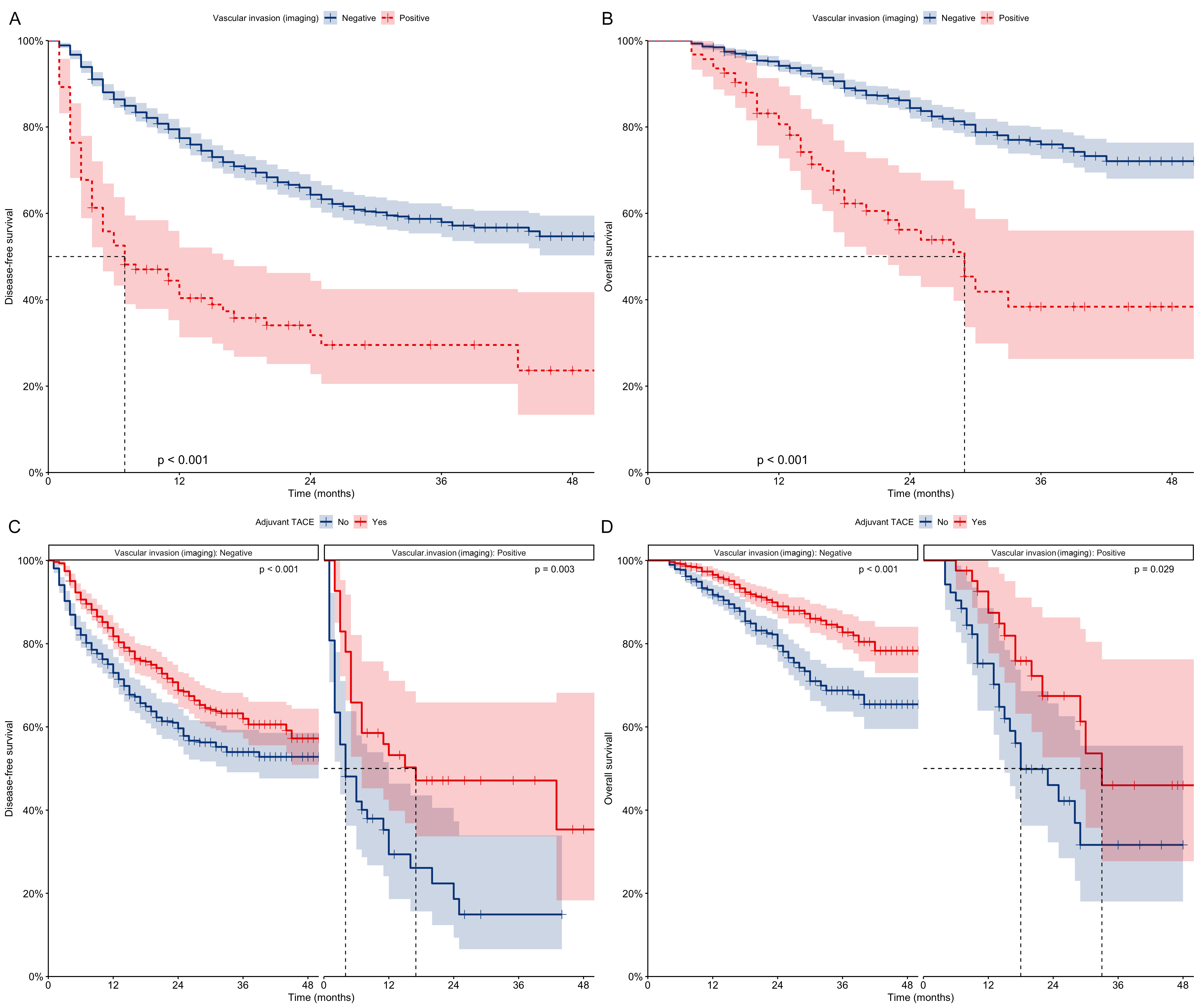

Supplement: Supplementary file 6 — Supplementary Material 6 [file 12885_2023_10802_MOESM6_ESM.tif]

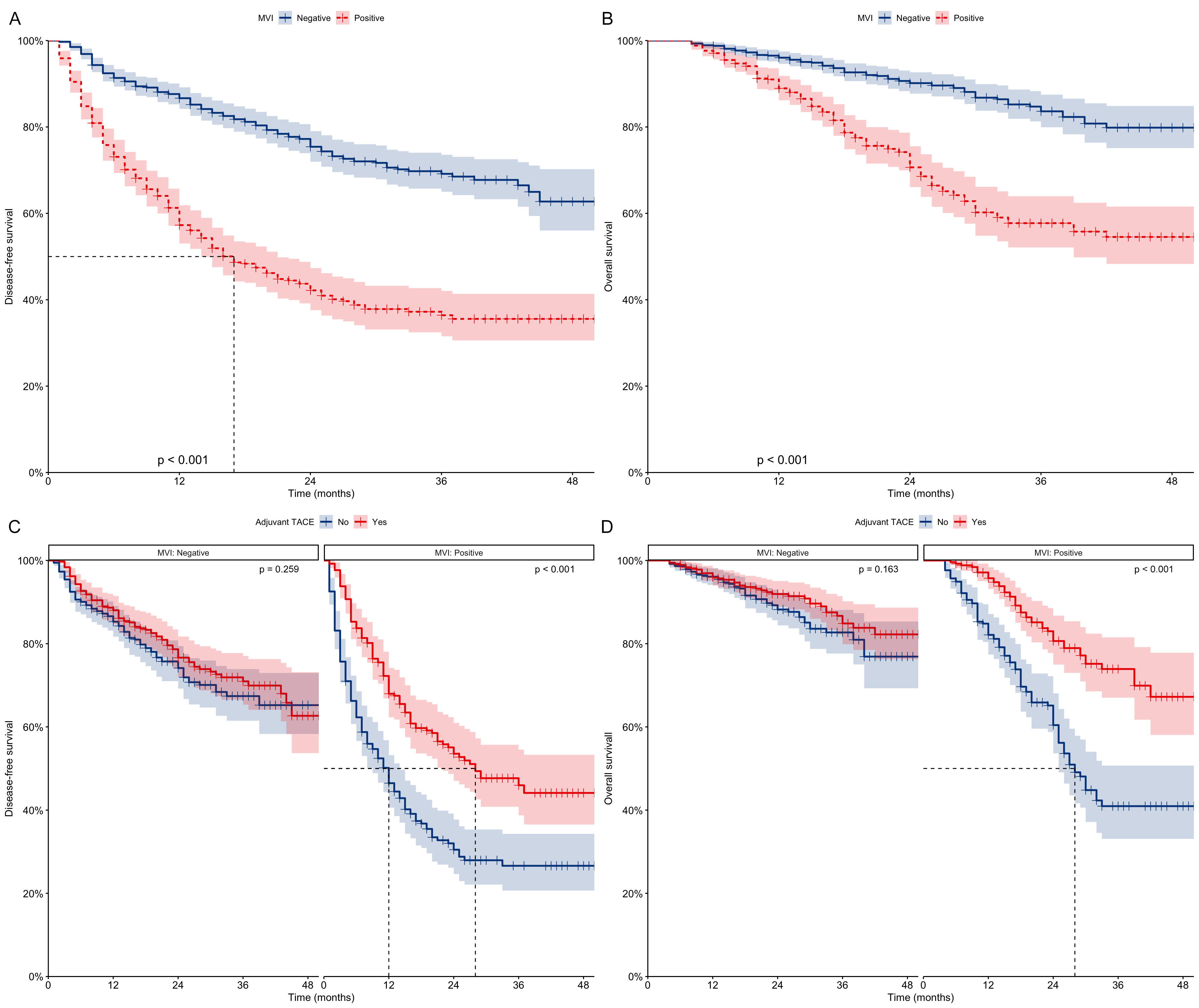

Supplement: Supplementary file 7 — Supplementary Material 7 [file 12885_2023_10802_MOESM7_ESM.tif]

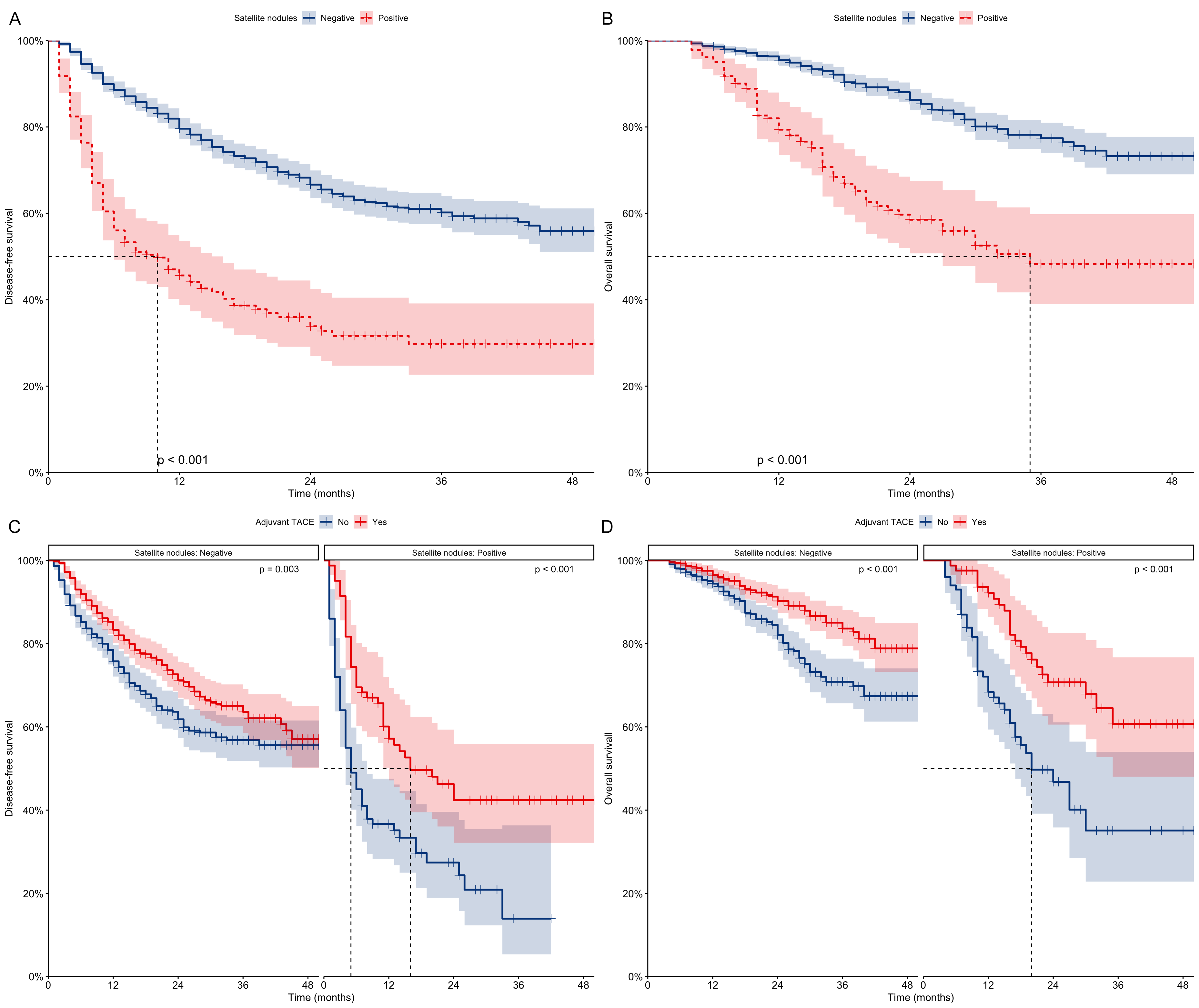

Supplement: Supplementary file 8 — Supplementary Material 8 [file 12885_2023_10802_MOESM8_ESM.tif]

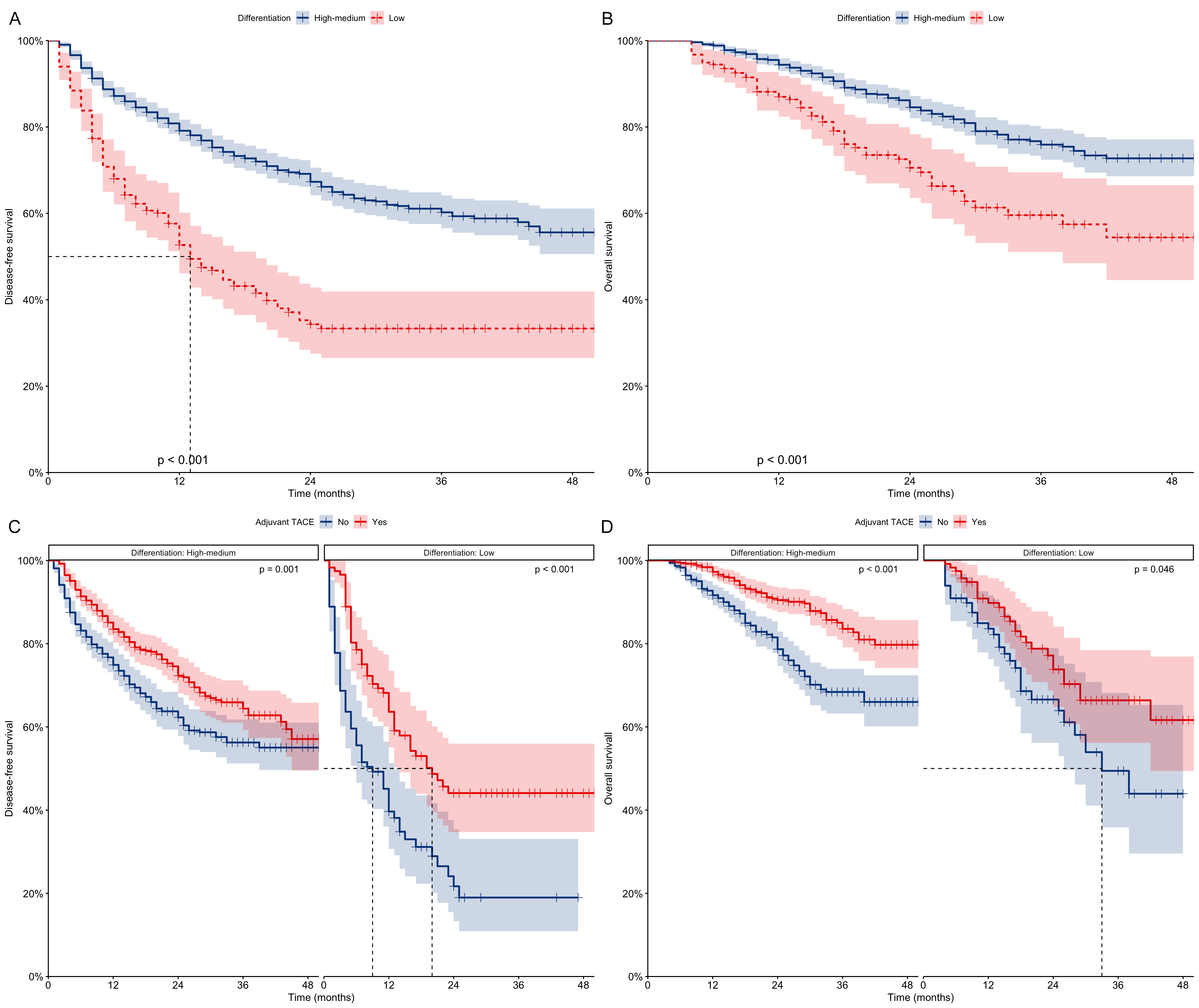

Supplement: Supplementary file 9 — Supplementary Material 9 [file 12885_2023_10802_MOESM9_ESM.tif]
