# Supplementary material for: Efficacy of adjuvant TACE on the prognosis of patients with HCC after hepatectomy: a multicenter propensity score matching from China
Source: BMC Cancer. 2023 Apr 7;23:325. doi: 10.1186/s12885-023-10802-9 (PMC10080834; doi:10.1186/s12885-023-10802-9)
Supplement: Supplementary file 10 — Supplementary Material 10 [file 12885_2023_10802_MOESM10_ESM.docx]

**Fig.S1**

Flow chart of the enrolled patients in our study.

**HCC**, Hepatocellular carcinoma; **CNLC**, Chinese liver cancer; **FAHNU**, The First Affiliated Hospital of Nanchang University ; **SAHNU**, The Second Affiliated Hospital of Nanchang University; **SPH**, Shenzhen People's Hospital; **ZPH**, Zhongshan People's Hospital; **TACE**, Transarterial chemoembolization

**Fig.S2**

Selection of cut-off point (Black dots) for AFP by triangular X-tile plot based on DFS (A);

DFS-based histogram with 996.7ng/mL as the cut-off value for AFP. The blue bar represents patients with AFP ≤ 996.7ng/mL, while the gray bar represents patients with AFP > 996.7ng/mL (B);

DFS-based Kaplan-Meier analysis with 996.7ng/mL as the cut-off value for AFP. The blue curve represents DFS for patients with AFP ≤ 996.7ng/mL, while the gray curve represents DFS for patients with AFP > 996.7ng/mL (C);

Kaplan-Meier analysis of DFS was performed using 996.7 ng/mL as the cut-off value for AFP (D);

Subgroup Kaplan-Meier analysis of DFS for patients with different levels of AFP who received adjuvant TACE or not (E).

**AFP**, Alpha-fetoprotein; **DFS**, Disease-free survival; **TACE**, Transarterial chemoembolization

**Fig.S3**

Selection of cut-off point (Black dots) for LMR by triangular X-tile plot based on DFS (A) and OS (D);

Histograms based on DFS (B, 2.7) and OS (E, 2.5) are classified into low LMR group, high LMR group according to the optimal cutoff values. The blue bars represent patients with low LMR, while the gray bars represent patients with high LMR;

Kaplan-Meier analysis of DFS (C, 2.7) and OS (F, 2.5) based on the optimal cutoff values of LMR. The blue curves represent patients with low LMR, while the gray curves represent patients with high LMR;

Kaplan-Meier analysis of DFS (G) and OS (H) was performed using 2.6 as the cutoff value of LMR;

Subgroup Kaplan-Meier analysis of DFS (I) and OS (J) for patients with different levels of LMR who received adjuvant TACE or not.

**DFS**, Disease-free survival; **OS**, Overall survival; **LMR**, Lymphocyte-to-monocyte ratio; **TACE**, Transarterial chemoembolization

**Fig.S4**

Selection of cut-off point (Black dots) for maximum tumor diameter by triangular X-tile plot based on DFS (A) and OS (D);

Histograms based on DFS (B, 55 mm) and OS (E, 57 mm) are classified into low-value group, high-value group according to the optimal cutoff values for maximum tumor diameter. The blue bars represent patients with low value of maximum tumor diameter, while the gray bars represent patients with high value of maximum tumor diameter;

Kaplan-Meier analysis of DFS (C, 55 mm) and OS (F, 57 mm) based on the optimal cutoff values of maximum tumor diameter. The blue curves represent patients with low value of maximum tumor diameter, while the gray curves represent patients with high value of maximum tumor diameter;

Kaplan-Meier analysis of DFS (G) and OS (H) was performed using 55 mm as the cutoff value of maximum tumor diameter;

Subgroup Kaplan-Meier analysis of DFS (I) and OS (J) for patients with different levels of maximum tumor diameter who received adjuvant TACE or not.

**DFS**, Disease-free survival; **OS**, Overall survival; **TACE**, Transarterial chemoembolization

**Fig.S5**

Kaplan-Meier analysis of DFS for patients with different number of tumors (A); Subgroup Kaplan-Meier analysis of DFS for patients with different number of tumors who received adjuvant TACE or not (B); Kaplan-Meier analysis of OS for patients with different Child-Pugh classifications (C) and liver cirrhosis (E); Subgroup Kaplan-Meier analysis of OS for patients with different Child-Pugh classifications (D) and liver cirrhosis (F) who received adjuvant TACE or not.

**DFS**, Disease-free survival; **OS**, Overall survival; **TACE**, Transarterial chemoembolization

**Fig.S6**

Kaplan-Meier analysis of DFS (A) and OS (B) for patients with vascular invasion (imaging); Subgroup Kaplan-Meier analysis of DFS (C) and OS (D) for patients with vascular invasion (imaging) who received adjuvant TACE or not.

**DFS**, Disease-free survival; **OS**, Overall survival; **TACE**, Transarterial chemoembolization

**Fig.S7**

Kaplan-Meier analysis of DFS (A) and OS (B) for patients with MVI; Subgroup Kaplan-Meier analysis of DFS (C) and OS (D) for patients with MVI who received adjuvant TACE or not.

**DFS**, Disease-free survival; **OS**, Overall survival; **MVI**, Microvascular invasion; **TACE**, Transarterial chemoembolization

**Fig.S8**

Kaplan-Meier analysis of DFS (A) and OS (B) for patients with satellite nodules; Subgroup Kaplan-Meier analysis of DFS (C) and OS (D) for patients with satellite nodules who received adjuvant TACE or not.

**DFS**, Disease-free survival; **OS**, Overall survival; **TACE**, Transarterial chemoembolization

**Fig.S9**

Kaplan-Meier analysis of DFS (A) and OS (B) in patients with different differentiation types;

Subgroup Kaplan-Meier analysis of DFS (C) and OS (D) for patients with different differentiation types who received adjuvant TACE or not.

**DFS**, Disease-free survival; **OS**, Overall survival; **TACE**, Transarterial chemoembolization
